# Adolescence then and now: a qualitative study on older adults’ perceptions of adolescence and factors influencing mental health in adolescents today

**DOI:** 10.1186/s12889-025-24437-x

**Published:** 2025-09-24

**Authors:** Charlotte Konradsdatter Aronsen, Therese Daleng Bjørnnes

**Affiliations:** Institutt for Helse og Omsorgsfag, UiT, Norges Arktiske Universitet, Havnegata 5, 9405 Harstad, Norway

**Keywords:** Nursing, Sociology, Life lived, Sense of coherence, Medicalization, Social media

## Abstract

**Background:**

Among Norwegian adolescents, there have been increases in self-reported psychological ailments, consultations with health services for psychological ailments, and the use of antidepressants. At the same time, the population of Norway is getting older and is in increasingly less need of assistance in old age, and the prevalence of mental disorders among healthy older adults is probably relatively low compared to younger age groups.

**Aim:**

This study aimed to explore older adults’ perceptions of adolescence and factors influencing mental health to increase nurse’s societal knowledge.

**Method:**

The study had a phenomenological exploratory design. We conducted semi-structured interviews with eight older adults ranging from 68 to 90 years of age and analyzed their perceptions of being adolescents using systematic text condensation.

**Results:**

The conditions of being an adolescent in the past and now are significantly different, and the mentality about mental health differ. The family structure has changed, and adolescents socialize in a different way with less face-to-face interaction. The digitalization of society may give adolescents illusions about what you can expect in life, and medicalization conveys the idea that there is a cure for everything.

**Conclusions:**

The analyzed perceptions of the participants support that medicalization, social media, and the societal structure are impacting the sense of coherence and mental health of adolescents today.

**Supplementary Information:**

The online version contains supplementary material available at 10.1186/s12889-025-24437-x.

## Introduction

Nurses work across all age groups and healthcare settings and the nursing profession encompasses a broader societal responsibility, and thus responsibility for the ones who are to lead our society into the future.

Norwegian adolescents have exhibited increases in eating disorders, anxiety disorders, stress disorders, depressive episodes, and depression, according to a report on mental disorders in Norway by the Institute of Public Health. Accordingly, there have been increases in self-reported psychological ailments, consultations with health services for psychological ailments, and the use of antidepressants. The report emphasizes the urgency of understanding the underlying causes and consequences of these developments, as well as identifying effective strategies to mitigate mental distress among adolescents [1].

At the other end of the age spectrum, the population of Norway is getting older and has less need for assistance in old age [2]. Research conducted by the Norwegian Institute of Public Health has shown no clear indication that depression or anxiety become more prevalent with age; rather, the opposite has been observed. The 2025 Norwegian National Public Health Report posits that the prevalence of mental disorders among healthy, home-dwelling older adults is likely lower than in younger age groups, although further research is warranted [3].

These findings suggest that while older adults are enjoying more years with good mental health, adolescents are increasingly affected by mental health challenges and the need for treatment. Physiologically, adolescents are in their physical prime; however, emotional and relational difficulties are prevalent due to ongoing brain development and the critical role of social learning during this life stage [4]. The adolescent brain remains neurologically immature, particularly in the prefrontal cortex, which exerts limited control over the amygdala. This neurobiological configuration renders adolescents more susceptible to intense emotional reactions under stress. Stressors—ranging from public speaking to social exclusion and bullying—can significantly impair learning and memory, thereby increasing vulnerability to mental health disorders. Elevated stress hormone levels are among the most significant risk factors for the development of mental illness [5]. Social media further complicates the mental health landscape for adolescents. Its pervasive and compelling nature, combined with adolescents’ limited capacity for self-regulation, has been linked to poor sleep quality, heightened stress, and negative mental health outcomes [6–8].

The media functions as a key driver in the process of medicalization. Medicalization is a sociological term that can be defined as a comprehensive and complex process that leads to the extensive use of medical expertise and terminology, to the extent that people’s lives are increasingly being defined as relevant for medical intervention and placed under the responsibility of health services [9, 10]. This is reflected in the expansion of diagnostic categories in the ICD-10, which now encompass a wide range of human traits, emotions, and behaviors [9]. Barsky’s paradox of health [11] further complicates the discourse. This phenomenon suggests that heightened public health awareness may lead individuals to become overly attuned to bodily sensations and cognitive states, potentially pathologizing normal variations in mental and emotional experiences [12].

Aaron Antonovsky’s salutogenic model offers a complementary perspective by focusing on factors that promote health rather than those that cause disease. Central to this model is the concept of Sense of Coherence (SOC), which encompasses the degree to which individuals perceive life as comprehensible, manageable, and meaningful [13]. SOC has been shown to be a critical determinant of perceived health, particularly mental health, and is especially relevant in understanding adolescents’ coping mechanisms [14–16].

Nurses, who work across all age groups and healthcare settings, play a pivotal role in health education and policy development. Their professional responsibilities extend beyond clinical care to include social advocacy and the application of sociological insights [17]. By engaging in sociological reflection, nurses can challenge prevailing societal norms, influence public attitudes, and foster new understandings of health phenomena. A sociological perspective equips nurses to recognize and address the social determinants of health and illness, thereby enhancing their capacity to advocate for systemic change [18].

This study aims to explore older adults’ recollections of adolescence several decades ago and to analyze these insights through a health-oriented lens. The objective is to deepen understanding of the factors influencing adolescent mental health today. What phenomena might explain the apparent disparity in mental well-being between older adults and today’s youth? The findings are intended to inform nursing practice and contribute to broader awareness among adolescents, parents, and health professionals regarding the social conditions shaping contemporary youth experiences.

## Method

### Design

This study was designed as a phenomenological study with an exploratory qualitative approach [19]. The aim was to gain a deeper understanding of older adults’ lived perceptions of being young, to explore what phenomena would emerge from their narratives and how these may shed light on the mental challenges faced by adolescents today. The first author conducted semi-structured individual interviews with eight older adults. The Consolidated Criteria for Reporting Qualitative Research (COREQ) guidelines for qualitative studies were applied to ensure transparency and rigor in the research process [20]."

### Context and recruitment

The data collection took place in October 2022. The purpose was to interview adults over 67 years of age to ensure a generation difference, and older adults with dementia or severe physical illness were excluded. Participants were purposefully selected among healthy older adults, based on the rationale that adolescents, from a physiological standpoint, generally represent a healthy population. This approach was intended to ensure a more balanced and comparable foundation between the two groups and therefore we recruited the participants from a hiking group for healthy older adults. The first author distributed information about the study and a consent form to 52 members of the group in person. The consent forms were left at the group’s meeting place, and those who wished to participate signed one and placed it in a closed box for collection by the first author. Eight people were willing to participate. The interviews were transcribed continuously throughout the data collection process. The sample size was deemed saturated [21] after the 6th interview, when we observed that the central themes began to repeat, and new data increasingly confirmed already established patterns. Saturation was considered achieved when additional interviews no longer contributed to new insights or variations in the descriptions of the phenomena.

### Participants

The participants consisted of seven women and one man ranging from 68 to 90 years of age. The first author and the participants had no knowledge of each other prior to the interviews.

### Data collection

Individual interviews were conducted according to an interview guide developed for the study (Additional file 1) (for examples see Table 1). The data collection took place in a building adjacent to the hiking group’s meeting place, and only the first author and the participants were present. An audio recorder was used to enable later transcription. The interviews had a duration of 49–92 min.

**Table 1:** Examples from the interview guide

Examples from the interview guide	
How did you experience being an adolescent when you were growing up?	
How do you think it is being an adolescent today?	
What do you think is different about being an adolescent today compared to when you were young?	
When one has lived a long life, one has encountered ups and downs. What do you think is normal to feel when experiencing loss in life?	
Was mental illness, or mental health, a topic that was talked about when you were young?	
How would you describe living a life?	
Can you tell me what you think happiness is?	
What expectations do you think adolescents have for their lives?	

### Ethical considerations

The study was carried out in accordance with the Helsinki Declaration’s ethical research principles [22]. Written informed consent was obtained from all participants. They were informed that they could withdraw from the study without reason and could freely contact the first author if necessary. The study was also carried out in line with privacy legislation and in accordance with the regulations of the author´s research institution [23, 24]. All personal information was de-identified during transcription. Our data collection and management strategy were assessed by Sikt (Norwegian Center of Research Data) (Ref. 807,146) [25].

### Data analysis

We used systematic text condensation to analyze the data [19]. In step one, both authors read through the material separately and recorded preliminary themes based on their overall impressions. Authors negotiated the preliminary themes and coincident preliminary themes, and *older adults’ perceptions of mental health*, *the human as a social being*, *growing up being safer before*, *normality or illness*, and *influence* were identified as code groups. While the coding process provided a structural overview of the data, a deeper emotional reading revealed rich layers of meaning in participants’ reflections. Their narratives were not only descriptive but also emotionally charged, often expressing nostalgia, concern, and subtle critique. These emotional undertones offer valuable insight into how older adults interpret their own adolescence in contrast to the perceived challenges faced by adolescents today. Table 2 presents participants’ statements along with examples illustrating the analytical process.

**Table 2:** Examples from the analysis

Meaningful units	Code group	Subgroup	Subcategory	Main category
“When I grew up, it was a foreign term; we didn’t know anything about mental health” (Informant 7) “One sees that most things pass. Most problems resolve themselves, and you shouldn’t worry too much about things that might happen, because mostly, the things you worry about don’t happen either” (Informant 1)	Older adults’ perceptions of mental health	A different mentality One becomes stronger from adversity Mental illness was kept hidden Realism	Mental health was an unknown concept	Different times
“We had to seek contact with other people. They don’t need to do that today. It’s like they have enough, and more than enough” (Informant 2) “They kind of lose that interaction with people around them and… And that’s kind of scary, because it’s through that… you learn through others that you associate with… And that computer or that phone, it’s kind of one-track and one-sided” (Informant 6)	The human as a social being	A non-digital world Social structure Family structure Human relationships	It is in relationships with others that social competence is created	The interpersonal aspect is significant
“On social media, they get a lot of knowledge that they don’t have the ability to sort out. It can be too much for their little heads sometimes, and that’s not healthy” (Informant 3) “The materialistic society… I think for adolescents, many… happiness is to be… the figure on top; everything is great. And then to have lots of money… I think is associated with happiness” (Informant 5)	Influence	Expectations of life Social media Materialism A medicine for everything Unrealism Adolescents have a lot of knowledge	Social media creates illusions	Being young in a digital world must be challenging

In step two, we coded meaning units in the data that could shed light on the research question under the code groups from the previous step. In step three, we sorted the meaning units into various subgroups, and a condensate was produced for each subgroup, in line with Malterud, as well as for each code group. The condensate was illustrated by what Malterud refers to as the “golden quote.” The fourth step was the synthesis, where we wrote an analytical text for each subgroup and code group based on the condensates, and the chosen golden quote would concretize the main finding. We then developed the code groups into the study’s findings with the subgroups as subcategories [19]. The analytical text is presented in the results section as an introduction under the main findings.

## Results

The analysis resulted in three main categories and seven subcategories, presented according to different levels of headings.

### Different times

Participants described an upbringing involving freedom, responsibility, and close family ties. Many recalled contributing to household tasks from a young age and being expected to manage challenges independently. It was common for grandparents to live in the same house or on the same farm, creating a closeness to life and death. There were stories of resilience based on freedom under responsibility and trial and error. They talked about challenges they grew up with: they were brought up to “face it” and experience for themselves that most things pass. Participants believe this strengthened them, and this also seemed like a source of pride. They described an inclusive upbringing in a small local environment, where friendships were established even with age differences. There was a culture of including others, and they used each other when they needed to talk to someone. Their narratives conveyed a deep emotional connection to a time when life was perceived as simpler, more grounded, and socially cohesive. Mental health was not a topic growing up; they never heard this term. They experienced that those who had mental disorders were instead labeled with insults and people who were mentally ill were feared.

#### Having to face it

Participants described their adolescence as standing on their own feet from an early age. They had freedom, but with that freedom came responsibility and expectations. The mentality they described was that they were brought up to handle the struggles they faced.

*We were not coddled; it was demanded that we be adults, that we should manage on our own. Expectations were set for us. I don’t know, because I never heard in my upbringing if I struggled and couldn’t manage, if things went against me, that it was a pity for me and that it should be... that it was... No, you just must get up and move on and forget what happened, forget the stupid things that happened. You can handle this; it’s nothing to worry about. We can’t worry about everything.* (Informant 3)

Some participants described accepting things for how they were and making the best of whatever situation: “I believe it strengthens your self-esteem. You need challenges to grow” (Informant 1). In other words, they viewed adolescents today as over-protected, and this could lead to less resilience.

#### Growing up in an inclusive community

All participants said that they grew up in small, safe communities where social inclusion was the norm, and their narratives were accompanied with gratitude and warmth.

*It was safe and good. And it wasn’t so big back then, so we knew people our own age and we knew their parents and we knew where they lived too. So, the local community was overall manageable and nice. And we could talk to someone.* (Informant 7)

They made friends with others in proximity to them regardless of age differences. They had no other choice if they were to have friends. They played and got to know each other and shared secrets and concerns. One participant described the experience of going alone to confirmation camp:

*I think about how well they included us when we arrived there. And actually... we were at a confirmation gathering and... and I have mentioned it as many times as I could afterwards. Because now that I’m an adult, I see that it was not a given that they would welcome us and include us in the adolescents’ group—us who came from all sorts of strange villages around, such country bumpkins who arrived. No, we were supposed to be part of it.* (Informant 3)

Some participants said there was a mentality to include everyone and give individuals who were shy a push to participate. The shy ones were included in the social adolescent community, and the participants thought this was important because humans are social beings.

#### Mental health was an unfamiliar term

Mental health is a term which none of the participants were familiar with when they were young. Several described how mental illness was stigmatized or hidden; “swept under the carpet” within the family. These narratives were sometimes emotional, particularly when participants reflected on relatives who suffered.

*He was known as [stigmatizing nickname] so everyone was afraid of him. And just think about what it was like for him to be ill and alone, knowing that everyone was afraid of you.* (Informant 6)

Some participants expressed thoughts about how horrible it must have been for those who were mentally troubled and did not receive help. Some participants spoke of mentally troubled relatives who were secretly admitted to sanatoriums. It was never discussed. According to some participants, it is a good thing that the focus on mental health has increased, because mentally troubled persons can get the help they need, although the focus can be exaggerated. This express concern for the mental health of adolescents today.

#### The interpersonal aspect is significant

The participants assumed that growing up today is difficult and expressed concern. The extended family that was present when they grew up is rarer, both parents are in full-time jobs, and everyday life is busy and full of activities. According to some participants, there is little time for upbringing, and there is less closeness between parents and children in the fast-paced society. Some thought that mobility in society can affect the parents’ ability to be present for their adolescent children. Participants described their parents and grandparents as imparting life wisdom and that they always had someone to talk to. They reflected upon the fact that divorced parents are more common today, which they thought can affect adolescents’ sense of roots and identity. Participants also expressed that the digital solutions today lead adolescents to have less physical contact with other people and thus lose an important part of life. The emotional undertone here is one of empathy and concern. Participants were not simply criticizing modern parenting but expressing a deep-seated fear that adolescents are missing out on essential relational experiences that foster emotional resilience and social competence.

#### Society and family structure have changed

The participants described changes they have seen in the family structure over time. The extended family is now rare, and divorces are common. Participants spoke with a sense of loss from the values and rhythms that they felt once defined family life.

*There was more calmness before. Now it’s more stressful. Parents both work; it’s like… they don’t have time to raise their young properly. (Informant 8).*

Participants said that they believe family ties are important for identity and a feeling of safety, this reflection may not be only a critique of modern parenting but conveys a longing for a time when raising children was a shared, community-based effort. The emotional tone across these reflections can be understood as a mix of nostalgia, concern, and a protective instinct toward younger generations. Participants were not necessarily idealizing the past but rather highlighting what they believe has been lost in the transition to a more individualistic and digitally mediated society.

#### Social competence is created in relationships with others

Participants described growing up communicating through physical contact between people, as opposed to the high degree of remote communication today. There was no telephone, much less a television providing images and influences from distant places. All participants expressed that adolescents today are missing something important because they are less physically together. Participants emphasized that their own social skills were shaped by being part of a community where they had to engage with others, resolve conflicts, and support one another. They deemed it very important to be able to look in each other’s eyes, read body language, and use one’s vocabulary.

*I believe that it is very important to interact with other people. I think that’s tremendously important. Because you learn a lot from that. That’s what you learn from. You can’t get everything from a screen.* (Informant 5)

Participants feared that digital communication was replacing the nuanced, emotionally rich interactions that build empathy and understanding. The emotional tone in the narratives showed a concern. Participants worried that without these formative experiences, adolescents may struggle to navigate relationships and manage emotional challenges. It was also a feeling of sadness, participants felt sorry for adolescents today because they felt they were losing something vital in life.

### Being young in a digital world must be challenging

Adolescents are knowledgeable, the participants said, and they have all the world’s information available at their fingertips. They themselves were ignorant of much until they became adults and stated that they were allowed to maintain a childish mind longer than today’s adolescents are.

*We don’t let the kids be kids long enough. Instead of making them safe and resilient, we make them insecure by giving them knowledge they’re not ready for* (Informant 3).

Participants expressed concern because they thought young people get a lot of information that they are unable to handle, and it can be frightening having the whole world so close. Although adolescents are enlightened, they become adults prematurely owing to everything available to them, said Informant 1, and “Since they are so enlightened, they become grownups in a way— ‘grown-ups’ in quotation marks.” Participant 3 said that there is too much focus on the individual’s problems today, and this has a reverse effect that can magnify the problems.

#### Life must sting a little: views on mental health and ailments

Participants shared stories of losses in life and how they had coped with them, including the loss of parents at a young age, the loss of children, and children having serious illnesses. Everyone’s life contains hardships and crises, according to them, they saw this as a natural part of living life and portrayed a remarkable emotional clarity.

*It’s, like, supposed to be a medicine for everything today, because there shouldn’t be anything that stings anywhere. But sometimes, life stings, and then you must wait until it stops stinging. But not everyone can tolerate the sting. Because it’s not supposed to sting. Everything should be good all the time.* (Informant 3)

Some participants indicated that adolescents today might have overly high expectations of life, happiness, and the ability to access help for every ailment. Participants shared their reactions to hardships and said that it is normal to be sad for a long time and to feel scared. Their reflections suggest a belief that emotional pain, while difficult, is also necessary for growth. These are aspects of being human and not necessarily depression or anxiety. Informant 4 offered a personal health-related view: “I only had a heart attack and have diabetes. Other than that, I am healthy! [laughter].” Another participant talked about the children she had lost but ended the story by empathizing with adolescents today because she imagined that it is a tough world to grow up in and showed both wisdom and quiet strength.

#### An enlightened society for better or worse

The participants were aware that most knowledge is accessible on smartphones today. One participant opined that all this information comes at a high price and expresses concern that constant exposure to idealized lifestyles may distort adolescents’ self-perception and expectations.

*Since everyone has a mobile phone, they have access to everything. And that’s probably not healthy. Even though they become very enlightened, they also become “adults” at an early age— “adults” in quotation marks—because there’s nothing that is hidden, nothing that is not accessible.* (Informant 1)

Enlightenment and media serving as a source of information also lead to individuals obtaining information about illness and ailments. While participants appreciated the progress in mental health discourse, they also highlighted the paradox of enlightenment: that knowing more does not always mean coping better.

Several participants thought that perhaps adolescents expect things to be easy because they see on social media that everyone can have everything.

*I think they believe everything should come easily. And when they discover that it’s not like that, I think that’s when they have problems. I imagine that’s when the problems come, when they discover that everything isn’t as they thought. It isn’t so simple; you have to give something too. And that is... I think that’s an important lesson. So, exactly that lesson, I am grateful that I received it. I got the lesson that I had to do something to... give something to get something back. I don’t think that’s stupid.* (Informant 8)

This reflection reveals a deep understanding of the psychological impact of unrealistic expectations. Participants suggested that the pressure to control one’s life and achieve perfection may contribute to anxiety and dissatisfaction. They contrasted this with their own upbringing, where unpredictability and acceptance of fate were more common. Participants expressed concern that adolescents are under tremendous pressure and influence today, especially regarding how they should look.

## Discussion

The aim of this study was to explore older adults’ perceptions of adolescence and discuss the study’s findings from a health perspective to increase the understanding of phenomena that can affect adolescents’ mental health today. The findings reveal a generational contrast shaped by cultural, social, and emotional shifts. Participants’ narratives were marked by a deep sense of pride in resilience, a strong connection to community, and concern for the emotional well-being of adolescents growing up in a digital and fast-paced society.

### Two fundamentally different societies

Our participants described growing up in a society with stigmatization, little knowledge, and secrecy regarding mental illness. People who needed help with mental issues did not receive help due to the limited focus on mental health. Many participants said that society is moving forward. Providing enlightenment and help to people with mental ailments is a good thing, because they have seen what can happen to those who do not receive any help. Yet they mentioned that being understood as sick can also make you sick or sicker—too strong a focus on ailments makes the ailments grow. This aligns with a medicalized society where human characteristics are classified as conditions [4, 9]. Some adolescents need a diagnosis to get the help they need, which participants supported. Nevertheless, this does not exclude that increased public concern about adolescents’ mental health and diagnoses of mental health problems can affect how the adolescents perceive themselves and how they are treated in the healthcare system. Society and the media seem to be reflecting little on this mental health paradox when it comes to attention to adolescents and mental health [11, 12]. Participants described their upbringing as being raised to face their challenges, and their experience was that they could overcome troubles themselves, and they believed that this made them more resilient. A lack of knowledge of mental health and ailments combined with being raised to handle what comes your way seemed to lead to a mentality in the older adults that feelings like sadness, anxiety, or fear are normal and a natural part of life. This contradicts today’s medicalized society with its focus on diagnosing and treating every ailment and could be interpreted as older adults having acquired resilience as protection from medicalization and the health paradox, and that they have a stronger SOC [26].

Participants warm memories of the safety in the extended family and sense of belonging contrast with their thoughts on how the family structure has changed today. Their narratives contained empathy, nostalgia, and a protective instinct toward younger generations, suggesting that the perceived erosion of these social foundations may have significant implications for adolescent mental health. Adolescence, from a salutogenic perspective, is a journey of developing one’s identity within a social reality that is understood and is linked to the three components of SOC: comprehensibility, manageability, and meaningfulness [26]. When everything is permitted and the choices are unlimited, adolescents are burdened. A close family structure with strong bonds of love, mutual dependence, and a clear identity, contributes to a stronger SOC [26]. The participants commented that the family structure has changed and moved away from the extended family structure, and they stated that this can affect adolescents’ mental health negatively, which studies also suggest [27–29]. That is, contemporary society is busy, and there is little time for parenting and upbringing, which affects adolescents today, and therefore parents perhaps make arrangements for the adolescents to avoid challenges they, as the parents, do not have the capacity to follow up on. As one participant said, they were not coddled like adolescents are today. The emotional tone in this reflection was one of pride, but also subtle critique. Participants expressed concern that adolescents today are overprotected, potentially limiting their opportunities to develop resilience. This generational difference in coping strategies may reflect broader societal changes in parenting norms and mental health discourse.

Participants emphasized the interpersonal as important for human life and that we learn through interacting face to face with others. They spoke of their upbringing as inclusive and social, involving friends they relied on sharing their troubles with. Participants’ emotional responses, ranging from sadness to worry, suggest that they view this shift not just as a technological change, but as a loss of something fundamentally human. Adolescents today are also social, but the way they are social has changed [6, 30]. Although adolescents’ social lives tend to exist on media platforms to a larger degree than before, research shows that activities undertaken as a family are connected to several positive life outcomes [31, 32], and this is in line with elements of the salutogenic perspective that contributes to a stronger SOC in adolescents [26]. Participants narratives point to—that erosion of social structures and interpersonal closeness may weaken adolescents’ SOC, making them more vulnerable to psychological distress and mental unhealth.

### Does media contribute to mental unhealth?

Today’s adolescents are the first generation that has been exposed to a staggering number of electronic distractions. Digital toys trigger a chain reaction of neural processes that activate the brain’s reward system and the secretion of dopamine. Experiments have been conducted in which several thousand teenagers from different countries were without any form of media for 24 h, and they reported serious stress reactions afterward [5]. Social media can cause stress in adolescents [33, 34], which is a negative factor for learning and memory, and predisposes them to mental disorders. It also affects adolescents’ ability to handle everyday life. Social media is part of adolescents’ everyday lives, and it impacts their mental health and well-being at a vulnerable time in life [5].

The digital society and social media can influence adolescents in various ways, and participants thought that adolescents face significant pressure today to have a perfect appearance and a materialistic view of life—and a belief that money can buy happiness and serve as medicine for every ailment. Furthermore, medicalization creates expectations of almost perfect health and a problem-free life, but these expectations are unrealistic [18]. Social media supports medicalization as a catalyst for unrealism, and for adolescents, it is difficult to see beyond this facade. When they compare themselves to unrealistic role models, they get false hopes for the way they are supposed to master their lives. Feelings of not mastering one’s own life and of not being pretty enough, smart enough, or good enough are self-destructive thoughts that can affect mental health and therefore individuals’ possibilities for self-expression and quality of life [35]. Participants in this study thought that adolescents today have high expectations for life and that they see money and success as prerequisites for happiness, and express both concern and sadness that this contributes to stress, insecurity and unrealistic expectations. With social media, the distinction between professional, adult actors and the influence of peers has become more blurred, and the ideals conveyed on social media can increase the pressure to appear successful and have a perfect appearance [36]. Seeing past this facade is difficult, and the odds of having symptoms of depression or other mental ailments are significantly higher for adolescents with more frequent use of electronic media [37–39]. Some participants expressed concern that the enormous amount of information accessible to adolescents is unhealthy and that they see things they are unable to process. The adolescent brain is receptive to manifold impressions [5]. According to one participant, there is too much information for the brain to handle and it causes internal chaos for adolescents. They themselves had learned “safe” information from their elders, but adolescents today are exposed to all the misery in the world.

Adolescents want to learn more about emotions in school. They want to learn about stress, how to handle life when it gets difficult, and specific skills to help them when they experience stress and anxiety [40]. This could be understood as today’s adolescents having too few tools to face a medicalized and media-driven society that feeds them concepts and ideals, and they lack the resilience that older adults acquired when growing up and have a weaker SOC. These factors contribute to poor mental health.

### Does society promote learnt helplessness instead of a sense of coherence?

“All must fit in the same box,” one participant said about adolescents, conveying that there is little room to be different today in comparison to before. Research shows that structural problems can become individual problems, and it is a well-known phenomenon where differences in developmental levels can be interpreted as mental illness. Children and adolescents’ cognitive abilities do not develop at the same pace, but in today’s school system, students are expected to display the same academic skills at the same time, despite individual developmental differences [4]. This is well documented in studies regarding the risk of attention-deficit/hyperactivity disorder (ADHD) in relation to birth month [41–43].

Moreover, depression can be linked to the experience of lacking autonomy and influence over one’s life situation, and psychologists have, therefore, advocated for a counterbalance to psychiatric diagnostic manuals and more of a focus on resources than on dysfunction [44]. Feeling a lack of control over one’s life is linked to a phenomenon called “learned helplessness,” where people have experienced that they were helpless in a situation and therefore lose the motivation to alter their circumstances even when an opportunity arises [45]. The focus on individual problems in the school system is in line with the medicalization in contemporary society. Medicalization as a force gives the impression that everything in life can be treated, even uncomfortable feelings, and that you are not responsible for your own life [46]. As a process, this can be understood as leading to a higher degree of learned helplessness, where the focus is on ailments and not on resources, and a contradiction in “having to face it.”

Antonovsky’s theory focuses on what brings health and can also be seen as a counterbalance to psychiatric diagnostic manuals. He explicitly highlights the responsibility of society to create conditions that induce the strengths of coping—that is, SOC. Society must shift its focus to resources and promote the mentality that adolescents are allowed to be different, instead of fitting into the same box, as one participant mentioned. This could contribute to a stronger SOC in adolescents [14].

### Methodological considerations

The study had a limited number of participants, but on a qualitative basis, the perceptions of the older adults were rich and useful. We have tried to reflect on the context to enable the reader to assess whether the results are transferable to a similar context. A weakness of the study may be the ages of the participants and the authors, which imply life experience that adolescents have not yet acquired, thus affecting the preunderstanding in the analysis process. Since the participants were predominantly female, this may have influenced the perspectives presented in the study. Gender can play a role in how perceptions are interpreted and expressed. Despite the participants being cognitively healthy, there remains a risk that their recollections of youth may be subject to inaccuracies. The transcripts were not returned to the participants, they could therefore not correct statements that might have been misinterpreted by the researchers and this is also a possible weakness of the study.

The concept of medicalization and sociological processes alone cannot explain the increase in the proportion of mental disorders among the young, but our interpretation of the findings still adds insight about adolescents and the effects on their mental health.

## Conclusion

Older adults’ perceptions of adolescence offer a unique and valuable perspective on the phenomena influencing adolescent mental health today, thus leading to increased nursing knowledge regarding societal structures that may affect our next generations. While this study does not establish causal relationships, the findings provide important empirical insights into the sociocultural dynamics that shape adolescents’ experiences. These reflections help to set to light why older adults may appear to possess a stronger sense of coherence (SOC) and a greater capacity to cope with life’s challenges.

By drawing on the lived experiences of Norwegian older adults, the study highlights key differences between past and present adolescence. It underscores how medicalization, social media, and structural changes in society influence adolescents’ mental health and their ability to develop a strong SOC. These insights suggest that today’s adolescents are growing up in a more complex and demanding environment—one that may undermine their resilience and emotional well-being.

From a nursing perspective, this knowledge is highly relevant. It can be used to challenge prevailing societal structures, expand the nurse’s educational and preventive role, and support adolescents in navigating their vulnerabilities. Nurses, as both caregivers and educators, are in a unique position to foster resilience and promote mental health by addressing not only individual needs but also the broader social conditions that affect youth.

We recommend further large-scale qualitative research on Norwegian adolescents’ mental health, with a particular focus on how to strengthen SOC in a media-driven and medicalized society. Such research could contribute to a deeper understanding of how societal expectations and structures either support or inhibit adolescents’ ability to cope. Ultimately, this knowledge can inform public health strategies and empower nurses to advocate for a society that better supports the mental health and development of its youngest members.

## Supplementary Information

Below is the link to the electronic supplementary material.

Supplementary Material 1

## Data Availability

The data material analyzed is available from the corresponding author on reasonable request.

## Abbreviations

SOC: sense of coherence
ICD-10: International classification of diseases

## References

[1] Reneflot AAL, Aase H, Reichborn-Kjennerud T, Tambs K, Øverland S. Psykisk helse i Norge [Mental health in Norway]. Folkehelseinstituttet/Norwegian institute of public health; 2023.

[2] Storeng SH, Øverland S, Skirbekk V, Hopstock LA, Sund ER, Krokstad S, Strand BH. Trends in disability-free life expectancy (DFLE) from 1995 to 2017 in the older Norwegian population by sex and education: the HUNT Study. Scand J Public Health. 2022;50(5):542–51. 33908292 10.1177/14034948211011796

[3] Bruun T, Denison EM-L, Gjersing L, Husøy T, Knudsen AKS, Strand BH. Folkehelserapporten - kortversjon. Helsetilstanden i Norge 2018 [Public health report - short version. Health conditions in Norway]. Folkehelseinstituttet, Område for psykisk og fysisk helse/Norwegian institute of public health, division for mental and physical health; 2023.

[4] Lunde C. Økende antall unge uføre med psykiske lidelser: en systemfeil eller en generasjonssvakhet? [Increasing number of young people on welfare with mental Disorders: a system failure or a generational weakness?]. Søkelys på arbeidslivet/Spotlight on working life. 2021;38(1):74–7.

[5] Jensen FE, Nutt AE, Lund B. Tenåringshjernen: hjerneforskerens overlevelsesguide til livet med ungdom [The teenage brain: a neuroscientist's survival guide to raising adolescents and youth]. Oslo: Pax; 2016.

[6] Hjetland GJ, Schønning V, Hella RT, Veseth M, Skogen JC. How do Norwegian adolescents experience the role of social media in relation to mental health and well-being: a qualitative study. BMC Psychol. 2021;9(1):78. 33985590 10.1186/s40359-021-00582-x PMC8120824

[7] Alonzo R, Hussain J, Stranges S, Anderson KK. Interplay between social media use, sleep quality, and mental health in youth: A systematic review. Sleep Med Rev. 2021;56: 101414. 33385767 10.1016/j.smrv.2020.101414

[8] Bakken A, Sletten MA, Eriksen IM. Generasjon prestasjon? Ungdoms opplevelse av press og stress [Generation achievement? adolescents’ experience of pressure and stress]. Tidsskrift for ungdomsforskning/Magazine of youth research. 2018;2:46–76.

[9] Markussen S, Røed K. Bidrar medikalisering av ungdom til utstøtning fra skole og arbeidsliv? [Does medicalization of young people contribute to exclusion from education and employment?]. Søkelys på arbeidslivet/Spotlight on working life. 2020;37(4):219–37.

[10] Hofmann BM. Too much technology. BMJ. 2015;350: h705. 25687230 10.1136/bmj.h705

[11] Barsky AJ. The paradox of health. N Engl J Med. 1988;318(7):414–8. 3340120 10.1056/NEJM198802183180705

[12] Ødegård G, Pedersen W. Ungdommen [Youth]. Oslo: Cappelen Damm Akademisk; 2021.

[13] Hochwälder J. Sense of coherence: notes on some challenges for future research. SAGE Open. 2019;9(2):2158244019846687.

[14] Eriksson M, Lindström B. Antonovsky’s sense of coherence scale and its relation with quality of life: a systematic review. J Epidemiol Commun Health. 2007;61(11):938–44. 10.1136/jech.2006.056028 PMC2465600 17933950

[15] Eriksson M, Lindström B. Antonovsky’s sense of coherence scale and the relation with health: a systematic review. J Epidemiol Commun Health. 2006;60(5):376–81. 10.1136/jech.2005.041616 PMC2563977 16614325

[16] Moksnes UK, Espnes GA. Sense of Coherence in Association with Stress Experience and Health in Adolescents. Int J Environ Res Public Health. 2020;17(9):3003. 32357461 10.3390/ijerph17093003 PMC7246660

[17] Yrkesetiske retningslinjer for sykepleiere [Professional ethical guidelines for nurses]: Norsk Sykepleierforbund; 2023 [cited 2024 31.01]. Available from: https://www.nsf.no/etikk-0/yrkesetiske-retningslinjer-sykepleiere.

[18] Ingstad K. Sosiologi: i helsefag og sykepleie [Sociology: in health sciences and nursing]. 2nd ed. Oslo: Gyldendal; 2021.

[19] Malterud K. Kvalitative forskningsmetoder for medisin og helsefag [Qualitative research methods for medicine and health sciences]. 4th ed. Oslo: Universitetsforlaget; 2017.

[20] Tong A, Sainsbury P, Craig J. Consolidated criteria for reporting qualitative research (COREQ): a 32-item checklist for interviews and focus groups. Int J Quality Health Care. 2007;19(6):349–57. 10.1093/intqhc/mzm042 17872937

[21] Hennink M, Kaiser BN. Sample sizes for saturation in qualitative research: a systematic review of empirical tests. Social Science & Medicine. 2022;292: 114523. 34785096 10.1016/j.socscimed.2021.114523

[22] Association WM. WMA Declaration of Helsinki – Ethical Principles for Medical Research Involving Human Subjects. 2018 [Available from: https://www.wma.net/policies-post/wma-declaration-of-helsinki-ethical-principles-for-medical-research-involving-human-subjects/. 19886379

[23] Alver BG. Ansvar for den enkelte [Responsibility for the individual] 2015 [cited 2024 25.01]. Available from: https://www.forskningsetikk.no/ressurser/fbib/personvern/ansvar-for-den-enkelte/.

[24] UiT Norges arktiske universitet. Informasjonssikkerhet og personvern ved UiT [Information security and privacy at UiT The Arctic University of Norway] s.a. [cited 2024 21.01]. Available from: https://uit.no/om/informasjonssikkerhet#kapittel_639070.

[25] Sikt. Personverntjenester for forskning [privacy services for research] Oslo: Sikt; [cited 2024 21.01]. Available from: https://sikt.no/tjenester/personverntjenester-forskning.

[26] Antonovsky A, Sjøbu A. Helsens mysterium: den salutogene modellen [The mystery of health: the salutogenic model]. Oslo: Gyldendal akademisk; 2012.

[27] Fransson E, Turunen J, Hjern A, Östberg V, Bergström M. Psychological complaints among children in joint physical custody and other family types: considering parental factors. Scand J Public Health. 2016;44(2):177–83. 26553250 10.1177/1403494815614463 PMC4735678

[28] Bergström M, Fransson E, Wells MB, Köhler L, Hjern A. Children with two homes: psychological problems in relation to living arrangements in Nordic 2- to 9-year-olds. Scand J Public Health. 2019;47(2):137–45. 29644929 10.1177/1403494818769173

[29] Juwariah T, Suhariadi F, Soedirham O, Priyanto A, Setiyorini E, Siskaningrum A, et al. Childhood adversities and mental health problems: a systematic review. J Public Health Res. 2022;11(3):22799036221106612. 36052096 10.1177/22799036221106613 PMC9425896

[30] Moberg KE, Vogt KC. Gutters tidsbruk på dataspill og skolearbeid [Time spent on computergames and homework in boys]. Nordisk Tidsskrift for Ungdomsforskning [Nord J Youth Research]. 2022;3(2):171–89.

[31] Walton K, Breen A, Gruson-Wood J, Jewell K, Haycraft E, Haines J. Dishing on dinner: a life course approach to understanding the family meal context among families with preschoolers. Public Health Nutr. 2021;24(6):1338–48. 32686634 10.1017/S1368980020001779 PMC10195635

[32] Miller DP, Waldfogel J, Han W-J. Family meals and child academic and behavioral outcomes. Child Dev. 2012;83(6):2104–20. 22880815 10.1111/j.1467-8624.2012.01825.x PMC3498594

[33] Song J, Yang J, Yoo S, Cheon K, Yun S, Shin Y. Exploring Korean adolescent stress on social media: a semantic network analysis. PeerJ. 2023;11: e15076. 36992939 10.7717/peerj.15076 PMC10042152

[34] Van Der Schuur WA, Baumgartner SE, Sumter SR. Social media use, social media stress, and sleep: examining cross-sectional and longitudinal relationships in adolescents. Health Commun. 2019;34(5):552–9. 29313723 10.1080/10410236.2017.1422101

[35] Primack BA, Shensa A, Sidani JE, Whaite EO, Lin LY, Rosen D, et al. Social media use and perceived social isolation among young adults in the U.S. Am J Prev Med. 2017;53(1):1–8. 28279545 10.1016/j.amepre.2017.01.010 PMC5722463

[36] Steinnes KK, Teigen HF. Sårbare bilder på sosiale medier: En studie om bildedelingskultur, kommersielle påvirkninger og kritisk medieforståelse blant ungdom [Vulnerable images on social media: a study on image sharing culture, commercial influences, and critical media literacy among adolescents]. Forbruksforskningsinstituttet SIFO, OsloMet/Institute for consumer research (SIFO), Oslo Metropolitan University; 2021.

[37] Kleppang AL, Steigen AM, Ma L, Søberg Finbråten H, Hagquist C. Electronic media use and symptoms of depression among adolescents in Norway. PLoS One. 2021;16(7):e0254197. 34234359 10.1371/journal.pone.0254197 PMC8263301

[38] Twenge JM, Campbell WK. Associations between screen time and lower psychological well-being among children and adolescents: evidence from a population-based study. Prev Med Rep. 2018;12:271–83. 30406005 10.1016/j.pmedr.2018.10.003 PMC6214874

[39] Twenge JM. More time on technology, less happiness? Associations between digital-media use and psychological well-being. Curr Dir Psychol Sci. 2019;28(4):372–9.

[40] Klomsten AT, Stenseng F. Behovet for å lære om egne følelser [The need to learn about one’s own emotions]. Bedre skole/Better School. 2019;31(1):55–61.

[41] Karlstad Ø, Furu K, Stoltenberg C, Håberg SE, Bakken IJ. ADHD treatment and diagnosis in relation to children’s birth month: nationwide cohort study from Norway. Scand J Public Health. 2017;45(4):343–9. 28482754 10.1177/1403494817708080

[42] Holland J, Sayal K. Relative age and ADHD symptoms, diagnosis and medication: a systematic review. Eur Child Adolesc Psychiatry. 2019;28(11):1417–29. 30293121 10.1007/s00787-018-1229-6 PMC6800871

[43] Vuori M, Martikainen JE, Koski-Pirilae A, Sourander A, Puustjaervi A, Aronen ET, et al. Children’s relative age and adhd medication use: a finnish population-based study. Pediatrics (Evanston). 2020;146(4):e20194046. 10.1542/peds.2019-4046 32958613

[44] Maier SF, Seligman MEP. Learned helplessness at fifty: insights from neuroscience. Psychol Rev. 2016;123(4):349–67. 27337390 10.1037/rev0000033 PMC4920136

[45] Nuvvula S. Learned helplessness. Contemp Clin Dent. 2016;7(4):426–7. 27994405 10.4103/0976-237X.194124 PMC5141652

[46] Lian OS. Når helse blir en vare: medikalisering og markedsorientering i helsetjenesten [When health becomes a commodity: medicalization and market orientation in health services]. Oslo: Cappelen Damm Akademisk; 2022.

