# Supplementary material for: Adolescence then and now: a qualitative study on older adults’ perceptions of adolescence and factors influencing mental health in adolescents today
Source: BMC Public Health. 2025 Sep 24;25:3060. doi: 10.1186/s12889-025-24437-x (PMC12462161; doi:10.1186/s12889-025-24437-x)
Supplement: Supplementary file 1 — Supplementary Material 1 [file 12889_2025_24437_MOESM1_ESM.docx]

**Interview Guide:**

**Introduction to the interview:**

Before we begin, I would like to know your age, which we will clarify and note before the interview starts.

Questions will be asked in a natural order based on how the conversation unfolds, and I will ask follow-up questions where it feels appropriate.

**Main Questions:**

How did you experience being an adolescent when you were growing up?

How do you think it is being an adolescent today?

What do you think is different about being an adolescent today compared to when you were young?

When one has lived a long life, one has encountered ups and downs. What do you think is normal to feel when experiencing loss in life?

Was mental illness, or mental health, a topic that was talked about when you were young?

How would you describe living a life?

Can you tell me what you think happiness is?

What expectations do you think the adolescents have for their lives?

**Follow-up questions:**

Could you describe some of the most common feelings or experiences you had during your adolescent years?

What words would you use to describe these reactions?

Do you think it would have felt different for you if these reactions had a name, like a diagnosis? Why or why not?

Did people generally have knowledge about mental illness/health?

What words were used when talking about mental distress?

When one has lived a long life, one has encountered ups and downs. What do you think is normal to feel when experiencing significant losses in life, such as losing someone close?

How do you perceive the mental health of youth today? Can you elaborate/explain. Why do you think it is so?

If I say that research shows that young people today use a lot of medication for depression and anxiety, what do you think about that?

**Ending of interview:**

Thank you so much for participating in the interview. Your perspectives and experiences are valuable to our understanding of the topic.
